# Extreme nonlinear strong-field photoemission from carbon nanotubes

**DOI:** 10.1038/s41467-019-12797-z

**Published:** 2019-10-25

**Authors:** Chi Li, Ke Chen, Mengxue Guan, Xiaowei Wang, Xu Zhou, Feng Zhai, Jiayu Dai, Zhenjun Li, Zhipei Sun, Sheng Meng, Kaihui Liu, Qing Dai

**Affiliations:** 1 Division of Nanophotonics, CAS Key Laboratory of Standardization and Measurement for Nanotechnology, CAS Center for Excellence in Nanoscience, National Center for Nanoscience and Technology, Beijing, 100190 China; 2 Center of Materials Science and Optoelectronics Engineering, University of Chinese Academy of Sciences, Beijing, 100049 China; 3 Beijing National Laboratory for Condensed Matter Physics and Institute of Physics, Chinese Academy of Science, Beijing, 100190 China; 4 Department of Physics, National University of Defense Technology, Changsha, 410073 China; 5 School of Physics, Academy for Advanced Interdisciplinary Studies, Collaborative Innovation Center of Quantum Matter, Peking University, Beijing, 100871 China; 6 Department of Physics, Zhejiang Normal University, Jinhua, 321004 China; 7 Department of Electronics and Nanoengineering, Aalto University, Tietotie 3, FI-02150 Finland; 8 QTF Centre of Excellence, Department of Applied Physics, Aalto University, FI-00076 Aalto, Finland

**Keywords:** Carbon nanotubes and fullerenes, Carbon nanotubes and fullerenes

## Abstract

Strong-field photoemission produces attosecond (10^−18^ s) electron pulses that are synchronized to the waveform of the incident light. This nonlinear photoemission lies at the heart of current attosecond technologies. Here we report a new nonlinear photoemission behaviour—the nonlinearity in strong-field regime sharply increases (approaching 40th power-law scaling), making use of sub-nanometric carbon nanotubes and 800 nm pulses. As a result, the carrier-envelope phase sensitive photoemission current shows a greatly improved modulation depth of up to 100% (with a total modulation current up to 2 nA). The calculations reveal that the behaviour is an interplay of valence band optical-field emission with charge interaction, and the nonlinear dynamics can be tunable by changing the bandgap of carbon nanotubes. The extreme nonlinear photoemission offers a new means of producing extreme temporal-spatial resolved electron pulses, and provides a new design philosophy for attosecond electronics and photonics.

## Abstract (web summary)

Strong-field photoemission is the emission of electrons driven by a strong electric field of light. Here the authors demonstrate a highly non-linear strong-field photoemission, approaching the 40th power-law scaling, from carbon nanotubes, yielding a photoemission current with up to 100% carrier-envelope phase modulation depth.

## Introduction

Strong-field photoemission occurs when the optical-field is sufficiently strong to bend the vacuum barrier at the material surface, and then a purely perturbative description of the emission process in the photon-driven picture is no longer sufficient^1–3^. In such a regime, electron emission is controlled by carrier waveform of the laser pulse rather than its envelope, and occurs in a fraction of an optical cycle^4–6^. By employing near-infrared or visible laser pulses, it is possible to generate electron pluses with an attosecond temporal resolution and with a high degree of synchronization to the incident optical waveform^7–13^. Not only does this advance time-resolved electron characterization into an attosecond time domain, but it also provides attosecond control and measurement methodology^14,15^. Strong-field photoemission thus lies at the heart of attosecond technologies such as attosecond electron microscopy^16^, peta-hertz electronic devices^17–19^, attosecond light sources^20,21^, and optical-phase detectors^15,22^, among others. The physical picture of strong-field photoemission has been established by the study of gas-phase targets, based on which attosecond light pulses and electron pulses have been achieved^11,13^. In recent years, the emerging strong-field physics at the nanoscale solids become a hot research topic, as it combines attosecond processes with nanoscales^23,24^. This not only brings a unique and sometimes unexpected strong-field phenomena due to the distinctive electronic structure of nanoscale solids, but is also promising for realizing highly integrated attosecond instruments and devices. In addition, the local field enhancement of nanoscale solids allows to access strong-field regime at a relatively low laser intensity.

As the optical-field strength (*F*) increases, photoemission may transit into a strong-field regime^8,25^, marked by the Keldysh parameter $\gamma = \omega \sqrt {2m\Phi } /e\beta F$ (*ω* is optical frequency, Φ is work function, *m* is the mass of the electron and *e* is its charge, and *β* is the field enhancement). When fully accessing a strong-field regime (*γ* < 1), the optical-field is strong enough to create a penetrable barrier, and electron tunneling takes place from states within the vicinity of the Fermi level^1^, which is also called as optical-field emission (OFE). Over the past decade, OFE has been nondestructively accessed in various specially engineered nanostructures under a wavelength down to visible light^23,24^, which is a milestone of attosecond physics. In the OFE regime, the photoemission current (*I*) approximately follows the static field-emission rate driven by the instantaneous value of the optical-field^26^. The static field-emission current is given by the Fowler–Nordheim form $I \propto F_{{\mathrm{dc}}}^2{\mathrm{exp}}(4\sqrt {2m\Phi ^3} /3\hbar eF_{{\mathrm{dc}}})$ (*F*_dc_ is the static electric field). In this framework, the *I–F* nonlinearity—defined as a slope (*K*) of log–log plot of the *I–F* curve, is decreasing with the *F* increasing. Consequently, when fully accessing the OFE regime, it has been frequently observed that the photoemission curves bend down and depart from the multiphoton form^25–30^, with a reduced *K* ≈2. Unfortunately, the decreased nonlinearity leads to a less sensitive carrier-envelope phase (CEP) modulation effect^27^—the fundamental of attosecond control and measurement technology. Thus, great efforts have been devoted to achieving highly sensitive CEP modulation of the electronics signal by using alternative methods, such as extracting the high-order part in the electron energy spectrum^8,31,32^.

**Fig. 1:**
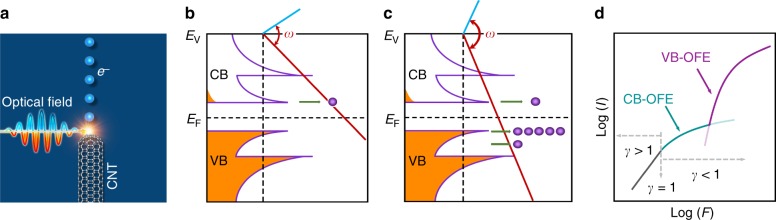
Operation principle of extreme nonlinear OFE. **a** Diagram of OFE from CNT. Electrons (blue balls) are emitted from semiconducting CNT, driven by a negative half-cycled (red line) strong electromagnetic field of a femtosecond laser. **b** Diagram of CB-dominated OFE (CB-OFE) that occurs at a relatively low optical-field strength. The electron-filling states (orange) in allowed bands (purple lines) follow the Fermi–Dirac distribution near Fermi level (*E*_F_). Red and blue lines represent the periodical vacuum level driven by a strong optical-field in negative and positive cycles, respectively, with a circular frequency of *ω*. Purple balls present the tunneling electrons. **c** Diagram of transition into VB-dominated OFE (VB-OFE) that occurs when the optical-field strength is increasing. **d** Illustration of the nonlinear photoemission *I–F* curve in log–log plot. The upward bending of the curve is a result of transition from CB-OFE (green line) to VB-OFE (red line). Both CB-OFE and VB-OFE follow the FN form (*γ* < 1). The difference in emission rates is induced by different electron amounts in CB and VB. Multiphoton photoemission occurs at a low driving field limit (*γ* > 1)

In this work, we demonstrate an extreme nonlinear photoemission behavior in the strong-field regime, by using semiconducting carbon nanotube (CNT) emitters^33,34^. In the case of one-dimensional (1D) CNTs (Fig. 1a), electron tunneling may start from conduction band (CB) states at a relatively low field due to the lower barrier height (Fig. 1b). However, the CB tunneling rate is limited by the low electron density in the CB. Near the edge of the valence band (VB), although the tunneling probability is much smaller than that at the CB, the electron density is much higher. As a result, the emission current from the VB edge can be comparable to or even larger than that from the CB (Fig. 1c). Therefore, the transition into VB tunneling emission may depart from CB tunneling emission, and behave as an upward bending of the *I*–*F* curve alongside with an extremely high *K*, as shown in Fig. 1d. The sharpness of the bending, referred to as the *K* of the *I–F* curve, should be proportional to the bandgap. The behavior has been observed in static-field emission from semiconductors^35^, which is expected to be observed in OFE in principle.

Indeed, we observe that the *I–F* curve bends up dramatically from the conventional OFE regime, with a greatly enhanced high nonlinearity (*K* ≈40). Then, we obtain a high CEP modulation depth up to 100%, with a high modulation current up to 2 nA, simply by using a few-cycle ultrafast oscillator and a source meter. The underlying mechanism of the behavior is believed to be an interplay of VB-OFE together with charge interaction, which is supported by quantum-mechanical time-dependent density functional theory (TDDFT) calculations and classical two-step Simpleman model calculations. Our measurements are informative for designs of future ultrafast, ultrabright nanostructured photocathodes, as well as extreme sensitive CEP detectors. From a more general perspective, our work takes a step toward improving temporal resolution of strong-field-driven, attosecond science and technology.

## Results

### Achieving semiconducting CNT cluster emitter

A single-wall CNT cluster, grown by chemical vapor depostion method, is employed as the emitter (see the “Methods” section for materials synthesis). The diameter of the cluster is 2 μm. Each CNT in the cluster has a sub-nanometric tip. By assuming a random distribution of CNT chiralities, one-third of CNTs in the cluster is metallic tubes, while others are semiconducting tubes^36^. Compared with semiconducting CNTs, the metallic ones have a superior OFE current, due to the higher electron density at their Fermi level. This will conceal the intrinsic OFE behavior of semiconducting CNTs. Therefore, to reveal the OFE behavior of semiconducting CNTs, the metallic ones must be removed from the cluster. In the present experiments, we applied an aging process—illuminate the emitter by a strong enough aging laser pulse to reach a current level beyond the threshold (saturation current), and then the metallic tubes will be removed due to overload, while the semiconducting tubes survive. A similar method has been successfully employed to remove metallic CNTs from CVD-grown horizontal CNT films for a semiconducting CNT transistor^37^. In the present work, the aging process opens the bandgap of the entire CNT cluster.

### Extremely nonlinear *I–F* curve

**Fig. 2:**
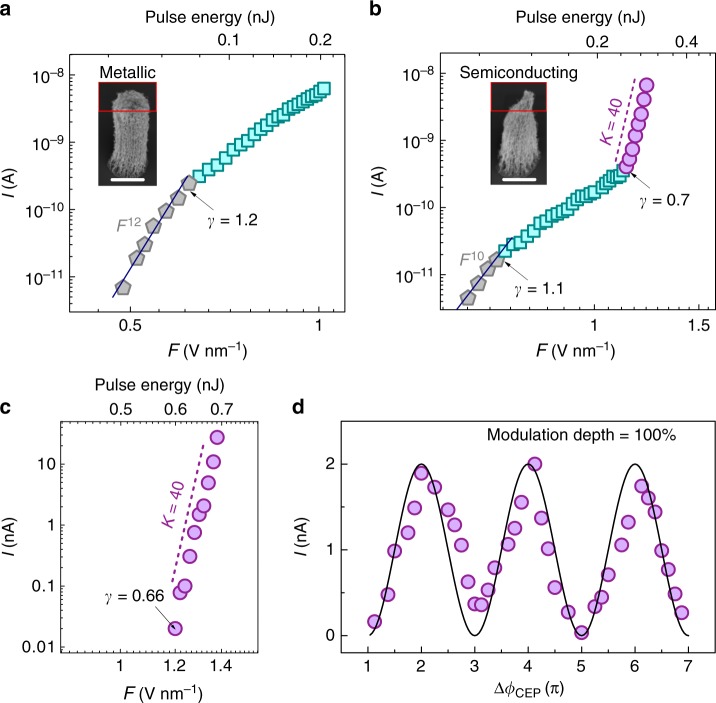
Extreme nonlinear OFE current. **a** Experimentally obtained log–log plot of optical-field (*F*) dependent total photoemission current (*I*) for a CNT cluster before aging, driven by 100-fs laser pulses with a central wavelength of 820 nm. Two linear behaviors are observed, as shown by different colors: multiphoton photoemission (MPP) (gray pentagonal dots); OFE (green square dots). The inset shows the scanning electron microscopy (SEM) image of the emitter. Scale bar is 2 μm. **b** *I–F* curve of aged CNT cluster in which the metallic tubes have been removed. Three linear behaviors are observed: MPP (gray pentagonal dots); CB-OFE (green square dots); VB-OFE (purple circular dots). An extremely high slope of *K* = 40 (dashed line) was observed. The inset shows the SEM image of the emitter. Scale bar is 2 μm. **c** CEP-stabilized measurement of the *I–F* curve of the same emitter, by using 7-fs few-cycle laser pulses centered around 800 nm. A slope of *K* = 40 is obtained as well. **d** The CEP-dependent photoemission current at a fixed laser intensity with a peak *F* = 1.3 V nm^−1^ with a cosine fit (solid line)

Typically, the *I–F* curves obtained by using 100-fs laser pulses with a central wavelength of 820 nm (see Methods for experiments), before and after applying an aging laser pulse with 1.4 V nm^−1^ optical-field strength, are shown in Fig. 2a, b. Before aging, the photoemission is believed to be dominant by metallic tubes, through which the *I–F* curve is shown in Fig. 2a. The inset shows the scanning electron microscopy (SEM) image of the as-grown CNT cluster. The low-field section of the curve can be fitted to multiphoton photoemission form^38,39^, *I* ∝ *F*^2*N*^. We find *N* = 6 for the as-grown CNT cluster (with a photon energy ℏ*ν* ≈1.55 eV). However, as the work function of CNT *Φ*_CNT_ ≈4.4 eV^33^, we expect *N* = 3. The previous experiment on gold nanotips has identified the similar increases, which were speculated to be attributed to either geometry effect or deeper energy band contribution^25^. With *F* increasing, the curve bends down and departs from the multiphoton form: the emission accesses the strong-field regime^27,29^. The departure occurs at *γ* ≈1.1 (calculated by using *β* ≈20 for CNTs, see Supplementary Fig. 1 and ref. ^34^). The behavior is similar to conventional findings from metallic nanostructures^25,26,29,30^.

The *I–F* curve of the aged CNT cluster emitter reveals a totally different photoemission behavior (Fig. 2b). The inset shows the SEM image of the aged CNT cluster, which shows a great shrink of the apex due to the laser annealing and removing of metallic tubes. At this stage, the photoemission is believed to be dominated by semiconducting tubes. Therefore, photoemission current is greatly reduced at the same optical-field strength, due to the greatly reduced electron density of states (DOS) near Fermi level. The *I–F* curve behaves as a conventional strong-field photoemission at low-field section (*F* < 1.1 V nm^−1^), with a transition from the multiphoton regime to a strong-field regime at *γ* ≈1.2. As expected, the *I*–*F* curve accesses a distinctive region at the *F* ≈1.1 V nm^−1^ (corresponding to *γ* ≈0.7), where the curve departs from the conventional OFE regime and bends up sharply. This behavior is believed to be the OFE from VB states of semiconducting emitter. In this unique OFE process, an ultrahigh photoemission nonlinearity is observed—the *I–F* curve shows extremely high *K* ≈40, which is much higher than all previous findings^25,26,29,30^.

### Highly CEP-sensitive photoemission

Having observed the extreme strong-field photoemission behavior, we next undertake the CEP-dependent photoemission measurements^26,27^. Ultrashort laser pulses of around three optical cycles (7-fs pulse duration) with an 800-nm central wavelength are used (see Methods for experiment details). The CEP-stabilized measurement of an *I–F* curve is shown in Fig. 2c. In this measurement, the total photoemission current is greatly reduced compared with the 100-fs case (Fig. 2b), due to a greatly reduced pulse width. Therefore, only the third region (VB-OFE regime) at the high *F* section in the *I–F* curve is clearly observed, in which *I* is above the noise current level (10^−11^ A). In the high *F* section (corresponding to *γ* <0.66), a similarly high nonlinearity (*K* ≈40) is observed. Then, we fixed the laser intensity (*F* ≈1.3 V nm^−1^), and controlled the CEP (Δ*ϕ*_CEP_) for photoemission measurements, as presented in Fig. 2d (see Supplementary Fig. 2 for more typical curves). As expected, in the extreme nonlinear VB-OFE regime, *I* is modulated effectively by changing the CEP^27^. By tuning CEP for 6*π*, the measured data points can be fitted to a Cosine curve quite well, providing solid evidence of a strong-field photoemission mechanism^8^. The modulation depth reaches up to 100%, which is around twice as high as previously reported values obtained by using metallic tips^27^. The total modulation current reached up to 2 nA, which is beyond the state-of-the-art techniques (around 3 pA)^26^. This result clearly shows that full access to the VB-OFE regime can offer more sensitive control of the photoemission process with CEP, which shows promise for a sensitive CEP detector simply by using a source meter, and then further improvement of the temporal precision of attosecond measurements and control^8^.

### Calculated electron dynamics by using TDDFT

**Fig. 3:**
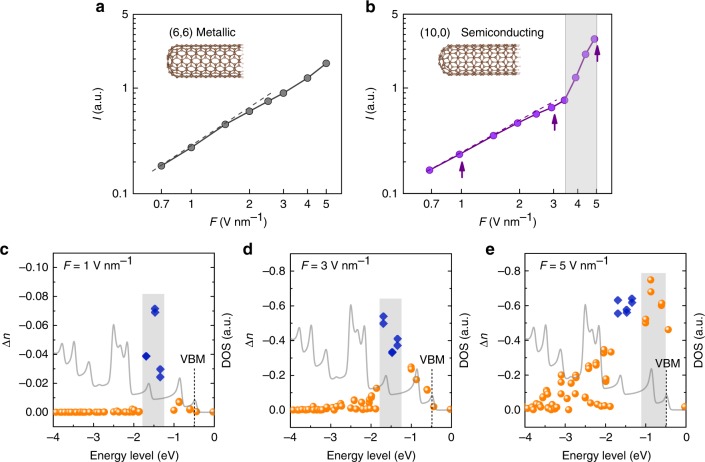
OFE behavior revealed by TDDFT calculation. **a** The calculated *I–F* curve of (6,6) metallic CNT model. **b** The calculated *I–F* curve of (10,0) semiconducting CNT model. The simulated laser pulse is centered around 800 nm (ℏ*v* = 1.55 eV) with a pulse width of 7 fs. MPP regime is indicated by a dashed line. Gray area indicates the curve bending up. **c**–**e** Excitation states of the semiconducting model (10,0) tube at three points marked by arrows in **b**. **c** At *F* = 1 V nm^−1^, the number (∆*n*) of excited electrons clearly show a peak at −1.5 eV (in gray area). Photon-driven electrons are represented by blue square dots, while field-driven electrons are represented by yellow circular dots. **d** At *F* = 3 V nm^−1^, the peak is unchanged, which also indicates a photon-driven dominated regime. **e** At *F* = 5 V nm^−1^, the peak moves to −0.9 eV, and the excitation number decreases rapidly as the energy level goes deeper, which demonstrates field-driven tunneling behavior. The DOS data are plotted as a gray solid line in (**c**–**e**). VBM is marked by the dashed line

After the demonstration of such extreme nonlinear strong-field photoemission behavior, we then theoretically depict the difference in photoemission behaviors from the metallic tube and semiconducting tubes. We calculate the photoemission process by using the framework of TDDFT, which covers both the photon (weak) and field (strong) regimes in a single description^40,41^. Calculations are performed for two single-walled CNT models—zigzag (10,0) semiconducting tube and armchair (6,6) metallic tube (see Supplementary Fig. 3a, b for DOS). The diameter of both models is smaller than 1 nm, which is within the range of tube diameters used in the experiments (see Supplementary Fig. 4c for Raman). An incident Gaussian light pulse with an 800-nm central wavelength and a full width at half maximum of 7 fs is used in our calculations. The photoemission current at the tips is calculated (see Methods). The calculated *I–F* curves of the two models are shown in Fig. 3a, b. We observe the expected photoemission behaviors for both metallic and semiconducting models. As shown in Fig. 3a, the curve can be fitted to a power-law scaling, which indicates a multiphoton photoemission regime. The curve bends down at high *F* section, which is an evidence of accessing the OFE regime. No curve bending up is observed in metallic models, which is consistent with all the findings in this work and literatures. As shown in Fig. 3b, the simulated curve of semiconducting model clearly shows an upward bending and deviation from conventional OFE scaling, which is consistent with the experimental observation in Fig. 2b.

Compared with the experimental results, the second part of the curve (CB-OFE regime) is much less prominent—the photoemission entered VB-OFE directly from photon-driven photoemission. The present model cannot reproduce the behavior of CB-OFE mainly because the CB is almost empty at low temperature. The laser heating effect may be involved in the experiments; however, this cannot be reproduced as only one laser pulse is simulated. In addition, it should also be noticed that the curve slope is much lower than the experimental results, which is because of a much smaller distance of the current-collection plane (only 0.2 nm, see Supplementary Fig. 3c) in the present model. The charge interaction, which is intrinsically considered in TDDFT, may greatly affect the calculated photoemission current. Due to the strong charge interaction at near field, the propagation of low kinetic energy electrons may be greatly suppressed. Therefore, at far field, the calculated results should be close to the experimental results. To confirm such a reason, we calculated the current at different plane distances, as shown in Supplementary Fig. 3d, which shows that the slope of the curve is exponentially increasing with the plane distance increasing. Further increasing the distance, the value of the slope may saturate to a constant that approaches the experimental result. However, this will cost a lot more calculation resources, and is impractical based on the current calculation ability. Besides, the realistic micrometer-long CNT contains much more electrons than the simulated model, which have more electrons accumulated at the tip area due to the “lightening rod effect”. This induces a much higher field enhancement factor, and thus contributes to the extreme-large slope observed in the experiment as well. As complements, we state three other methods to count the emitted electrons (see Supplementary discussion of the TDDFT simulation results and Supplementary Figs. 5–7).

As speculated above, the curve bending up is due to the electron tunneling from VB states. To examine this speculation, we calculated the excitation behaviors by tracking the changes of electrons in different energy levels at different ***F*** in three different regimes pointed by arrows in Fig. 3b (see Methods). With a lower optical-field (*F* = 1 V nm^−1^, Fig. 3c), the electrons are mainly excited from the states around an energy level of −1.5 eV, which is far from the valence band maximum (VBM) at −0.5 eV. This difference indicates a photon-driven regime, which occurs to satisfy the momentum conservation^42,43^. As the optical-field increases (*F* = 3 V nm^−1^, Fig. 3d), the number of photon-driven electrons increased greatly (almost eight times) with unchanged peak position (−1.5 eV). This suggests that excitation is still dominated by photon-driven regime. However, it is noticed that the energy spread of the excited electron broadens a little, which suggests that the strong-field effect starts to affect the excitation. With the higher optical-field (*F* = 5 V nm^−1^, Fig. 3e), the excitation shows a totally different behavior. The increasing of photon-driven electrons is largely suppressed, while the excitation peak moves to the energy level (−0.9 eV) near VBM, with excitation number reducing greatly when the energy level goes deeper. This clearly indicates that the excitation is dominated by the field-driven tunneling. In the field-driven tunneling emission regime, the tunneling (excitation) probability of electrons is far less dependent on the photon energy; rather it mainly relies on both the initial energy level and its DOS. The higher energy level, the larger tunneling probability, as it faces a narrower tunneling barrier. This is why the peak moves toward the VBM. However, tunneling probability is also limited by the DOS. This is why the excitation peak is not located exactly at the VBM. These results confirm that the observed *I–F* curve bending up in the present work is a result of strong optical-field-driven electron tunneling from the VB of semiconductors.

### Calculated electronic structure-dependent photoemission

The repeatability of the strong-field photoemission behavior is confirmed by investigating tens of CNT cluster emitters (see Supplementary Fig. 8 for more typical curves). However, we noticed that the slope of the third regime (VB-OFE) varies from sample to sample (24 < *K* **≤** 40 in the present work). As speculated above, the sharpness of the curve bending, referred to as *K*, is proportional to the bandgap. Thus, we believe that the *K* variation is due to the difference in the bandgap of the entire CNT cluster after aging process. To illustrate this variation, we compute the OFE current by using the extended two-step Simpleman model^1^ (see Methods). In the first step—electron tunneling, the computation is based on the integration of instantaneous Fowler–Nordheim (FN) tunneling current and considers all potential emissions from the occupied states near the Fermi level. In the second step—electron acceleration, the photoelectron starts with zero velocity at the position of the tunnel exit in the space-dependent (exponentially decays from the tip) ponderomotive potentials^8^. The deeper energy-level electron tunneling covers a longer distance and then gains less kinetic energy. In the present model, we set an energy threshold (*E*_th_) that the emitted electrons with kinetic energy smaller than *E*_th_ cannot be collected. The validity of this condition is evidenced by bias voltage-dependent photoemission measurement and kinetic energy spectra (see Supplementary Methods and Supplementary Fig. 9). There is a sharp increase in photoemission current upon increasing the bias from 25 to 90 V (Supplementary Fig. 9a). The fact suggests that part of the emitted electrons with the lowest kinetic energy cannot be collected (be driven back to the surface by Coulomb repulsion^30^, see Supplementary Discussion of charge interaction) at such low bias. The effect may vanish by increasing the bias voltage. However, this leads to a damage of CNT due to current overload. The value of *E*_th_ can be estimated by the electron kinetic energy spectra. In the spectra, the number of electrons with kinetic energy smaller than *E*_th_ should be greatly suppressed. As shown in Supplementary Fig. 9b, the cutoff energy at the low-energy side is around 1.5 eV, which suggests *E*_th_ ≈1.5 eV.

**Fig. 4:**
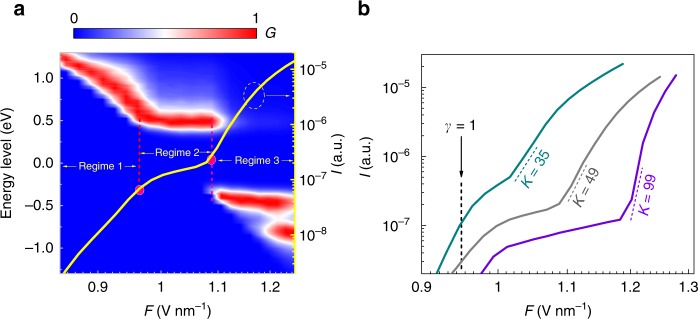
Illustration of bandgap dependency by Simpleman model calculation. **a** Contour plot of the *F-*dependent number (*G*, normalized at each *F* point) of emitted electrons from different energy levels, for a CNT model with a bandgap of ~1.0 eV. *I–F* curve (solid yellow line) is plotted. Transition points between different regimes are marked by red points. A transition point at *F* ≈1.1 V nm^−1^ is noted, before which the electrons mainly emit from CB states (above zero energy level), after which the electrons mainly emit from VB states (below zero energy level). At the same point, an upward bending of the *I–F* curve occurs. **b** *I–F* curves of three CNT models with different bandgaps: left (cyan)—0.5 eV, middle (gray)—1.0 eV, and right (purple)—1.5 eV. Higher bandgap is associated with greater nonlinearity. Note that the FN model-based simulation is only valid when *γ* < 1, which is the OFE regime. Here, we plot a longer *F* range to clearly show the transition from CB-OFE to VB-OFE. The multiphoton photoemission is not considered in low driving field limit (*γ* > 1) in the present calculations

Based on this model, the OFE behaves in three regimes, as shown in Fig. 4a—the contour plot of the *F*-dependent number of emitted electrons from different energy levels. The *I–F* curve is also plotted in the same figure. In Regime 1—low driving field limit, with the *F* increasing, electrons emitted from more and more deeper energy levels in CB will be collected. This leads to an extremely high slope of the *I–F* curve. In the second regime—medium driving field limit, as there are no electron states in the bandgap, the curve slope decreases. In Regime 3—high driving field limit, electrons emitted from VB gained enough kinetic energy (>1.5 eV) and thus can be collected, which leads to a steep increasing of slope again. It is clear that the bending up of the *I–F* curve occurs at the transition into VB electron emission regime, which is consistent with the above speculation.

Furthermore, the calculated *I–F* curves of the CNT models with different bandgaps (0.5, 1.0, and 1.5 eV, see Supplementary Fig. 10) are shown in Fig. 4b (see Supplementary Discussion and Supplementary Figs. 11–13 for more information). As expected, the slope of the curve (in the VB-OFE regime) increases with the bandgap increasing, while *I* decreases as a trade-off. Although *I* can be compensated by an enhanced *F*, it is limited by the finite mechanical strength of the CNT. Consequently, the photoemission nonlinearity, referred to as the modulation efficiency, must be balanced against the photoemission current for future applications. For larger-bandgap cases (1.0 and 1.5 eV), abrupt slope decreasing is noticed when the *I–F* curve transits from Regime 1 to Regime 2. This is due to the van Hove singularities (VHS) at the CB minimum, where the DOS drastically decreases to zero (see Supplementary Fig. 14). Note that the Simpleman model can only reproduce the OFE regime (*γ* < 1). In reality, in low driving field limit (*γ* > 1), the multiphoton photoemission current should be much higher than optical-field emission current, which will make the saturation smoother.

## Discussion

In conclusion, an extreme nonlinear strong-field photoemission with a curve slope of up to 40 is achieved for a CNT emitter, leading to a highly sensitive (up to 100%, modulation current up to 2 nA) CEP modulation. TDDFT calculation results show that the extreme nonlinearity is a result of optical-field-driven electron tunneling from the VB of the semiconducting CNT. Furthermore, such nonlinearity can be efficiently tuned by engineering the band structures of the emitter, which may be realized by controlling the chirality and doping levels of the CNTs. The concept is believed to be universal for other nanomaterials with tunable band structures. In future, the band-structure dependency on strong-field photoemission can be further explored in a platform of individual CNT device with controlled nanoscale tunneling gap (<10 nm). We predict that such highly sensitive optical-field control of the electron motion in nanostructures is a promising platform for the design of quantum electronics, and may pave the way for the generation, measurement, and application of attosecond electronics and optics.

## Methods

### Growth and characterization of CNT emitters

Vertically aligned single-walled CNT cluster arrays were grown on a highly doped n-type silicon chip by chemical vapor deposition (CVD). The silicon substrate was first coated with an Al (10 nm)/Fe (1 nm) multilayer catalyst, patterned by photo-lithography and deposited by sputtering. The substrate was then heated to 900 °C, at 10^–2^ mbar. During heating, gaseous ammonia was introduced to etch the surface of the catalyst and stimulate the formation of nanoislands. Acetylene was chosen as the carbon feedstock, and introduced to the deposition chamber once the temperature reached 900 °C. The growth process lasted for 1 minute, resulting in 10-μm-tall CNT clusters of defined areal patterns. Following the growth process, the samples were annealed in hydrogen at 1000 °C for 2 h to remove amorphous carbon deposits along with other remaining impurities and adsorbates. SEM image of the as-grown CNT cluster array is shown in Supplementary Fig. 4a. The high-resolution transmission electron microscopy (HRTEM, FEI Tecnai F20) result is shown in Supplementary Fig. 4b. The diameters of the CNTs were assessed from their radial breathing mode frequency (*ω*_RBM_ = 248/d (cm^−1^ nm^−1^)) through Raman spectrum^44,45^, as shown in Supplementary Fig. 4c. The pristine SWNTs were dispersed in absolute alcohol via ultrasonication and transferred onto a SiO_2_/Si substrate. Raman spectra were acquired by using He–Ne laser (632.8 nm) excitation, with data recorded by using a confocal micro-Raman spectrometer (HORIBA JobinYvon, LabRam HR 800) with 0.35 cm^−1^ resolution by using 1800 g mm^−1^ grating. The system had a 1-μm optical probe diameter by using a 100 × (N.A. = 0.9) Nikon objective.

### Measurements by using 100-fs laser system

Refer to ref. ^34^ for the experimental setup. Photoelectron emission from CNT arrays was triggered with 100-fs (Supplementary Fig. 15a and Supplementary Note 1) laser pulses, with a central wavelength of 820 nm, at an 80-MHz repetition rate from a Ti:Sapphire ultrafast laser (Spectra-Physics, Mai Tai-Series, SHG). A standard Si photodiode power sensor (Thorlabs S130C) was used to measure the laser power. White light and a charge-coupled device (CCD) were employed to monitor the sample position and the laser spot profile. The laser was linearly polarized with its polarization angle being controlled via a polarizer and a half-wave plate, as required. The laser was normally incident on the emitter via front illumination, which was focused to a 2.50-μm-diameter spot at the CNT cluster apex. To confirm that the CNTs are in the focus, we first check it by microscopy to move the CNT cluster to the center of the laser spot. Then, we carefully tune the laser power to obtain a moderate emission current. After that, we slightly scan the position of the CNT sample on the piezo stage (with 10-nm resolution) to obtain position with a maximum emission current, which we believe that the CNT emitter is right in the focus. Although the clusters contain many nanotubes, the growth kinetics were such that a few individual tubes protruded, repeatedly between growths, from these clusters producing a nanoscopic apex, which we believe is the main photoemission site giving the extremely high field enhancement. These photocathode samples were mounted in a high-vacuum chamber (10^−7^ Torr). The anode was isolated with the photocathode by using a thick mica-insulating spacer. The anode, together with the insulating separator, was placed directly on the surface of the photocathode with the CNT arrays centrally aligned. A Keithley 6430 source measurement unit was used to bias the anode with voltages of up to 50 V, with the anode current measured. Such a small static voltage generates a negligible effect on electron emission. All experiments are carried out in a high-vacuum chamber. Unless otherwise stated, the current measurements presented in this paper are those recorded at the anode. Every current data, collected by source meter, were acquired from an arithmetic average of 100 repeated measurements.

### Measurements by using 7-fs laser system

The setup is shown in Supplementary Fig. 16. A laser oscillator (Femtolaser CEP4) with a repetition rate of 75 MHz, centered at ~800 nm, a Fourier-limited pulse duration of <7 fs (Supplementary Fig. 15b and Supplementary Note 2). The spectral dispersion is managed with chirped mirrors and thickness of the materials in the beam path. The laser pulses are focused on the apex of the CNT clusters by an off-axis spherical mirror to an ~10-μm-diameter spot (Supplementary Fig. 15c), which can easily cover the apex of our CNT cluster, with an incident angle of ~45°. The laser spot was centered by measuring the position- dependent photoemission current. In such a setup, the photoemission current is proportional to the peak optical-field that is inversely proportional to the pulse width. Thus, to confirm that the pulse duration is almost the shortest at the sample, we measured the photoemission current at different thickness of the glass sheet. The pulse width is the shortest when the photoemission current reached the maximum. The carrier-envelope offset frequency *f*_CEO_ of the laser pulse is stabilized with an *f*–2*f* interferometer. Long-term drifts in the CEP are corrected by using an out-of-loop *f*–2 *f* interferometer. The optical-field induced photoemission current from the CNT cluster is measured by a Keithley 6430 source measurement unit with current preamplifier unit, which has a atto-ampere precision. The CEP of those pulses was controlled with a pair of fused silica wedges. Every current data, collected by the source meter, were acquired from an arithmetic average of 100 repeated measurements. All the data points of the *I–F* curves are obtained at a very stable situation (see Supplementary Fig. 17), which avoids the materials damage-induced photoemission current degradation.

### TDDFT computation method

The TDDFT simulations are done in two main steps. First, ground-state information of the CNT model is obtained based on density functional theory (DFT) calculations, including the optimized atomic structures as well as the corresponding electronic structures. Subsequently, real-time TDDFT approaches are employed to perform accurate simulations of the interaction between optical-field and CNT models, which is the state-of-the-art methodology. The *I–F* curves and the underlying electronic excitation dynamics are revealed without prior assumptions. The detailed calculation processes are introduced as follows.

Then we calculate the structure and ground-state properties of CNT models based on DFT. Calculations are performed for the semiconductor (10,0) and metallic (6,6) single-walled CNTs with one of the ends capped. Due to the absence of periodic boundary conditions in molecular calculations, it is necessary to saturate the carbon-dangling bonds with hydrogen atoms, yielding a C_200_H_10_ tube. The density functional theory (DFT) was performed with the Vienna Ab initio Simulation Package (VASP)^46^ to obtain the ground-state properties, by using a projector-augmented wave (PAW) pseudopotential in conjunction with the Perdew–Burke–Ernzerhof (PBE) functional4 and plane-wave basis set with energy cutoff at 400 eV. The atomic structure of the tube was positioned in a cubic supercell with vacuum regions of ~15 Å along three directions and fully relaxed until the force on each atom was <0.01 eV Å^−1^. The calculated DOS indicates that the nanotube behaves like a semiconductor with a gap of ~0.65 eV. As for a 1D periodic (10,0) carbon nanotube, the k points are sampled on a uniform grid along the tube axis (*N*_k_ = 150). We adopt a supercell geometry so that the tubes are aligned in a cubic array with the closest distance between adjacent tubes being 15 Å. At such a separation, the tube–tube interactions are very small so that they can be treated as independent entities. Other parameters are the same as those mentioned above.

Then we simulate the interactions between the optical-field and CNTs by real-time TDDFT. Real-time TDDFT represents a generalization of DFT to time-dependent systems^47–49^. TDDFT methods could directly provide time-domain evolution of electronic wave functions together with ionic movements, representing a versatile way for real-time tracking of ultrafast dynamics and phenomena either in perturbative or non-perturbative regimes. Therefore, it has been a unique ab initio quantum method applicable for the exploring of strong-field physics beyond linear response theory, for instance, high harmonic generation and ultrafast photoelectron emission. To date, a number of implementations of TDDFT have been reported with applications to both molecular and solid-state systems. In this work, two codes within the framework of TDDFT are used, i.e., OCTOPUS employing real-space grids and time-dependent ab initio package (TDAP) based on local atomic basis. Both of them describe the laser-matter interactions via solving the time-dependent Kohn–Sham (TDKS) equation:

1 $${\mathrm{i}}\frac{\partial }{{\partial t}}\varphi _i\left( {{\mathbf{r}},t} \right) = \left[ { - \frac{1}{2}\nabla ^2 + v_{{\mathrm{ext}}}\left( {{\mathbf{r}},t} \right) + v_{{\mathrm{Hartree}}}\left[ n \right]\left( {{\mathbf{r}},t} \right) + v_{{\mathrm{xc}}}\left[ n \right]\left( {{\mathbf{r}},t} \right)} \right]\varphi _i\left( {{\mathbf{r}},t} \right)$$

2 $$n\left( {{\mathbf{r}},t} \right) = \mathop {\sum}\nolimits_i^{{\mathrm{occ}}} {\left| {\varphi _i\left( {{\mathbf{r}},t} \right)} \right|^2}$$

Here, *φ*_*i*_(**r**,*t*) are the single-particle KS states (also called KS orbitals), *v*_ext_(**r**,*t*) is the time-dependent external potential that is generated by a optical-field, *v*_Hartree_[*n*](**r**,*t*) is the Hartree potential that describes the classical mean-field interaction of the electron distribution, *v*_xc_[*n*](**r**,*t*) is the exchange–correlation (xc) potential that includes all nontrivial many-body contributions, and *n*(**r**,*t*) is the many-body electronic density. Note that when the external field *v*_ext_(**r**,*t*) is zero, the TDKS equation will reduce to the ground-state KS equation. In our simulations, we use the length gauge to describe the interactions between the optical-field and 1D single-wall CNTs, where the vector and scalar potential of the field *E*(*t*) are ${\vec{\mathbf{A}}}\left( t \right) = 0$ and *v*_ext_ = −*E*(*t*)*z*, respectively. The applied external field is polarized along the axial direction (coordinate *z*) of the single-wall CNTs, and is shaped as a Gaussian pulse

3 $$E\left( t \right) = F\cos \left( {\omega t} \right){\mathrm{exp}}\left[ { - \frac{{\left( {t - t_0} \right)^2}}{{2\sigma ^2}}} \right]$$

The width *σ* is 3.5 fs, *ω* = 1.55 eV (*λ* = 800 m), and the laser reaches the maximum intensity *F* at the time *t*_0_ = 15 fs.

Then we simulate the tunneling current. OCTOPUS uses a real-space grid discretization to represent fields such as the Kohn–Sham states and the electronic density^50^. Each function is represented by its value over an array of points distributed in real space. Differential operators are approximated by high-order finite-difference methods, while integration is performed by a simple sum over the grid point coefficients. One of the main advantages of the real-space grid approach is that the discretization of Kohn–Sham states *φ*_*i*_(**r**,*t*) is more visually intuitive than any other basis such as plane-wave or atomic-orbital basis sets. The quality of the discretization can be increased by reducing the spacing and increasing the size of the simulation box, at the cost of an increased computational cost. In our calculations, the simulation grid is defined by assigning a spherical zone around each atom with a radius of 6.0 Å and a spacing of 0.2 Å. The selected simulation parameters can make a good balance between the calculation precision and cost. Meanwhile, the adiabatic local density approximation (ALDA) is used for the exchange–correlation functional, and Troullier–Martins pseudopotentials are used to represent the interaction between valence electrons and the atomic core. In our model, the photoemission is described by propagating electron wave packets {*φ*_*i*_(**r**,*t*)} under excitations for 28,000 steps, with a time step of 0.002 fs (Eq. (1)). During the simulation, a *sin*^2^ imaginary potential is added at the boundaries to improve the quality of the spectra by avoiding the formation of standing density waves. Then, the time- and space-dependent microscopic current density

4 $$j\left( {{\mathbf{z}},t} \right) = - \frac{{i\hbar }}{2}\mathop {\sum}\nolimits_k {\left\{ {\varphi _i^ \ast \left( {z,t} \right)\nabla \varphi _i\left( {z,t} \right) - \varphi _i\left( {z,t} \right)\nabla \varphi _i^ \ast \left( {z,t} \right)} \right\}}$$

is integrated across a chosen plane that is perpendicular to the *z* direction with a distance of 2.0 Å from the cap of the nanotube.

5 $$I\left( t \right) = \int_s {j\left( {{\mathbf{z}},t} \right) \cdot d{\mathbf{S}}}$$

The frequency-dependent current across this plane is obtained by taking the Fourier transform$I\left( \omega \right) = {\int} {dt\,I\left( t \right)e^{i{\mathrm{\omega t}}}}$. Then, the maximum intensity *I*(*ω*) is recorded with the increase in *F*.

Then we calculate the time-revolved changes in the occupation of KS states. We characterize the overall excitation dynamics through tracking the changes in the number of electrons in different energy levels. This part was performed with the TDAP as implemented in SIESTA^51–53^. Compared with real-space grids, the adoption of overwhelmingly efficient atomic-orbital basis sets are small in size and fast in performance, which enable simulations of a finite- size system with large vacuum space without heavy calculation cost while maintaining a relatively high accuracy.

To understand different behaviors of electrons under various field strengths, we project the time-dependent Kohn–Sham states, *φ*_n_(*t*), onto the ground-state (***t*** = 0) Kohn–Sham states, *ϕ*_m_ = 0, where *n* and *m* are the state indices labeled by increasing orbital energy

6 $$P_{{\mathrm{nm}} = }\left| {\left\langle {\phi _{\mathrm{m}}\left| S \right|\varphi _{\mathrm{n}}} \right\rangle } \right|^2$$

where *S* is the overlap matrix expressed with numerical atomic-centered orbitals. In this scenario, real-time excited state trajectories are achieved with many-electron densities self-consistently propagating at every electronic step, offering a direct microscopic picture on the ultrafast dynamics of electrons upon photoexcitation. The laser waveform is set in the same way as the previous step; however, a much larger time step of 0.05 fs is set to accelerate the computing process. The projection is performed at 27 fs.

### Simulation based on extended Simpleman model

We adopt the two-step Simpleman model^54^ to simulate the current density and electron kinetic energy. The diagram of the simulation model is shown in Supplementary Fig. 18a. The first step is the tunneling of electrons. In the quasi-static approximation, the emission probability at time *t* can be written as^55,56^

7 $$P_{{\mathrm{em}}}\left( t \right) = {\int} {P\left( {E,t} \right)dE}$$

8 $$P\left( {E,t} \right) = {\mathrm{DOS}}\left( E \right)f\left( E \right){\cal{T}}\left( {E,F_{z = 0}\left( t \right)} \right)$$

where DOS(*E*) is the energy density of states of the model, $f\left( E \right) = \frac{1}{{1 + {\mathrm{exp}}\left[ {E/\left( {k_{\mathrm{B}}T} \right)} \right]}}$ is the Fermi–Dirac distribution function at temperature *T* (*k*_B_ is the Boltzmann constant), *F*_*z* = 0_(*t*) is the oscillating electric field force at the tip (z = 0), and ${\cal{T}}\left( {E,F} \right)$ is the tunneling probability. For convenience, the Fermi energy is set as the zero energy. Our expression on *P*_em_(*t*) is equivalent to the formula of emission current in ref. ^13^ (Eq. (7) therein).

To determine the tunneling probability ${\cal{T}}\left( {E,F_{z = 0}\left( t \right)} \right)$, it is necessary to obtain the total electric field *E*_tot_(*z*,*t*) induced by the incident laser pulse ${\cal{E}}_{z = 0}\left( t \right) = F_{z = 0}\left( t \right)/( - e\beta )$. Here *z* is the distance to the tip, *β* is the field enhancement factor, and *e* is the fundamental charge. A simplified expression^1^ is adopted for the spatial variation of *E*_tot_(*z*,*t*)

9 $${\cal{E}}_{{\mathrm{tot}}}\left( {z,t} \right) = {\cal{E}}_{z = 0}\left( t \right)\left[ {\left( {\beta - 1} \right)\left( {\frac{{r_0}}{{r_0 + z}}} \right)^3 + \exp \left(\frac{{ - 2{\mathrm{ln}}2 \times z^2}}{{4w_{{\mathrm{foc}}}^2}}\right)} \right]$$

Here *r*_0_ is the tip radius and *w*_foc_ is the beam waist. The peak strength and 1/*e* decay length of the total field are $E_{{\mathrm{tot}}}^{{\mathrm{max}}} = \beta E_0$ and *l*_F_ ≈0.4*r*_0_. For CNT tips, the field enhancement varies with the radius as *β* = 24(*r*_0_[nm])^−0.5^. The electric potential energy created by the electric field ${\cal{E}}_{{\mathrm{tot}}}\left( {z,t} \right)$ is given by

10 $$\begin{array}{l}V\left( {z;F_{z = 0}\left( t \right)} \right) = {\int}_0^z {e{\cal{E}}_{{\mathrm{tot}}}\left( {z\prime ,t} \right)dz\prime } \\ = \frac{{F_{z = 0}\left( t \right)}}{{ - \beta }}\left[ {\frac{{\beta - 1}}{2}r_0z\frac{{z + 2r_0}}{{\left( {r_0 + z} \right)^2}} + \sqrt {\frac{{2\pi }}{{{\mathrm{ln}}2}}} w_{{\mathrm{foc}}}{\mathrm{erf}}\left( {\frac{{\sqrt {0.5\,{\mathrm{ln}}2} z}}{{w_{{\mathrm{foc}}}}}} \right)} \right]\end{array}$$

where ${\mathrm{erf}}\left( x \right) = \sqrt {\frac{2}{\pi }} {\int}_0^x {e^{ - \eta ^2}d\eta }$ is the Gauss error function. The tunneling probability is calculated from the Wentzel–Kramers–Brillouin (WKB) approximation^57^

11 $${\cal{T}}\left( {E,F} \right) = {\mathrm{\Theta }}(F){\mathrm{exp}}\left( - \frac{{2\sqrt {2m} }}{\hbar } \int_0^{z_E} {\sqrt {V\left( {z;F} \right) + {\mathrm{\Phi }} - E} } dz\right)$$

In this expression, Θ(*x*) is the Heaviside step function, *m* is the mass of a free electron in vaccum, ℏ is the reduced Planck constant, and Φ = 4.4 eV is the work function for CNT. The electric field force *F* at the tip causes electron emission only when it points outward the surface [*F* > 0 so that *V*(*z*;*F*) < 0 for *z* > 0]. The exit position *z*_E_ for the tunneling electron with energy *E* is determined by *V*(*z*_E_;*F*) + Φ = *E*.

The incident laser pulse has a time dependence

12 $${\cal{E}}_{z = 0}\left( t \right) = \frac{{F_{z = 0}\left( t \right)}}{{ - e\beta }} = - {\cal{E}}_0\cos (\omega t + \phi ){\mathrm{exp}}( - 2{\mathrm{ln}}2 \times t^2/\tau ^2)$$

where ${\cal{E}}_0$, *ω*, *ϕ*, and *τ* are, respectively, the peak strength, circular frequency, carrier-envelope phase, and pulse duration of the incident optical-field. At any time with *F*_*z* = 0_(*t*) > 0, we should calculate numerically the exit position *z*_E_ and then the tunneling probability for every possible initial energy *E*. This time-consuming calculation is avoided when the vacuum potential barrier for field emission can be approximated by a triangular-shaped barrier^56^ [*V*(*z*;*F*) = −*Fz*] so that

13 $$z_{\mathrm{E}} = \frac{{{\mathrm{\Phi }} - E}}{F}$$

14 $${\cal{T}}\left( {E,F} \right) = {\mathrm{\Theta }}\left( F \right)\exp \left[\frac{{ - 4\sqrt {2m} }}{{3\hbar F}}\left( {{\mathrm{\Phi }} - E} \right)^{\frac{3}{2}}\right]$$

The second step in the Simpleman model is the ponderomotive acceleration of emitted electrons under the total electric field ${\cal{E}}_{{\mathrm{tot}}}(z,t)$. For an electron with initial energy *E* emitted at time *t*_B_, the equation of motion $\ddot z\left( t \right) = - e{\cal{E}}_{{\mathrm{tot}}}(z,t)/m$ is numerically integrated by means of a fourth-order Runge–Kutta method with the initial position *z*(*t*_B_) = *z*_E_ (see Supplementary Fig. 18b for the typical exit position) and velocity *ż*(*t*_B_) = 0. Rescatterings with the tip are treated as perfect elastic collisions with a reflection probability *R* (*R* = 1 for full rescattering). From the evaluated single-particle trajectory, we obtain the final velocity *v*(*t*_B_,*ϕ*) and kinetic energy $\varepsilon _{\mathrm{K}}\left( {t_{\mathrm{B}},\phi } \right) = \frac{m}{2}[v\left( {t_{\mathrm{B}},\phi } \right)]^2$ which depend on the emission time *t*_B_ and carrier-envelope phase *ϕ*.

In the present model, we set a kinetic energy threshold *E*_th_ = 1.5 eV where the emitted electrons with *ε*_K_(*t*_B_,*ϕ*) < *E*_th_ or *v*(*t*_B_,*ϕ*) < 0 cannot be collected. The current density *j* of collected electrons is determined by the instantaneous generation probability, final kinetic energy, and kinetic energy threshold *E*_th_

15 $$j \propto {\int} {dt_{\mathrm{B}}} {\int} {dE\,P\left( {E,t_{\mathrm{B}}} \right){\mathrm{\Theta }}[\varepsilon _{\mathrm{K}}\left( {t_{\mathrm{B}},\phi } \right) - E_{{\mathrm{th}}}]{\mathrm{\Theta }}[v\left( {t_{\mathrm{B}},\phi } \right)]}$$

The kinetic energy spectra *F*(*ε*) of the final electrons are

16 $$F\left( \varepsilon \right) \propto {\int} {dt_{\mathrm{B}}} {\int} {dE\,P\left( {E,t_{\mathrm{B}}} \right){\mathrm{\Theta }}\left[ {\varepsilon _{\mathrm{K}}\left( {t_{\mathrm{B}},\phi } \right) - E_{{\mathrm{th}}}} \right]{\mathrm{\Theta }}\left[ {v\left( {t_{\mathrm{B}},\phi } \right)} \right]} \frac{{\frac{{\mathrm{\Gamma }}}{\pi }}}{{\left[ {\varepsilon - \varepsilon _{\mathrm{K}}\left( {t_{\mathrm{B}},\phi } \right)} \right]^2 + {\mathrm{\Gamma }}^2}}$$

where Γ is the energy resolution.

Here, we do a single active electron propagation and that this could in principle be extended by performing a many-electron simulation that includes the Coulomb interaction.

## Supplementary information

Supplementary Information

## Data Availability

The data that support the findings of this study are available from the corresponding authors upon reasonable request.

## Code availability

The code that was used to simulate the findings of this study is available from the corresponding authors upon reasonable request.
