# Supplementary material for: Extreme nonlinear strong-field photoemission from carbon nanotubes
Source: Nat Commun. 2019 Oct 25;10:4891. doi: 10.1038/s41467-019-12797-z (PMC6814826; doi:10.1038/s41467-019-12797-z)
Supplement: Supplementary file 1 — Supplementary Information [file 41467_2019_12797_MOESM1_ESM.pdf]

Supplementary Information

# Extreme Nonlinear Strong-field Photoemission from Carbon Nanotubes

Li *et al.*

**Supplementary Figures**

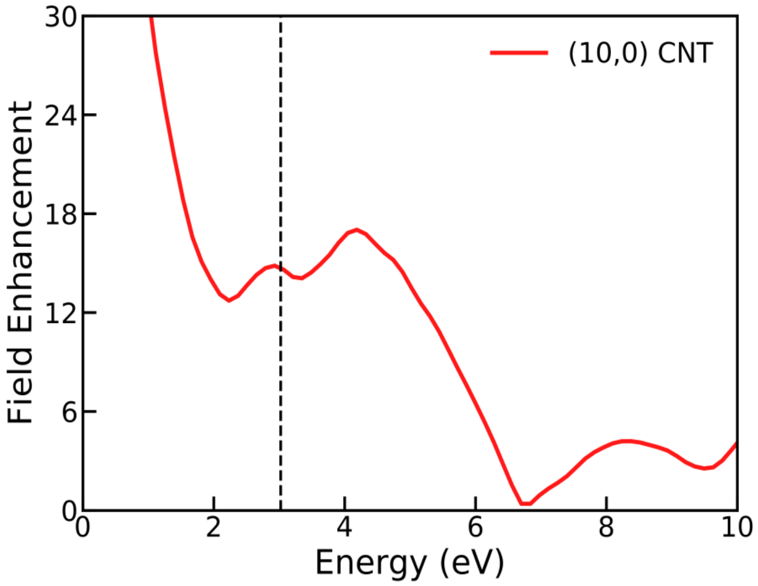


**Supplementary Figure 1.** Calculated field enhancement along the axis of (10, 0) CNT as a function of photon energy.

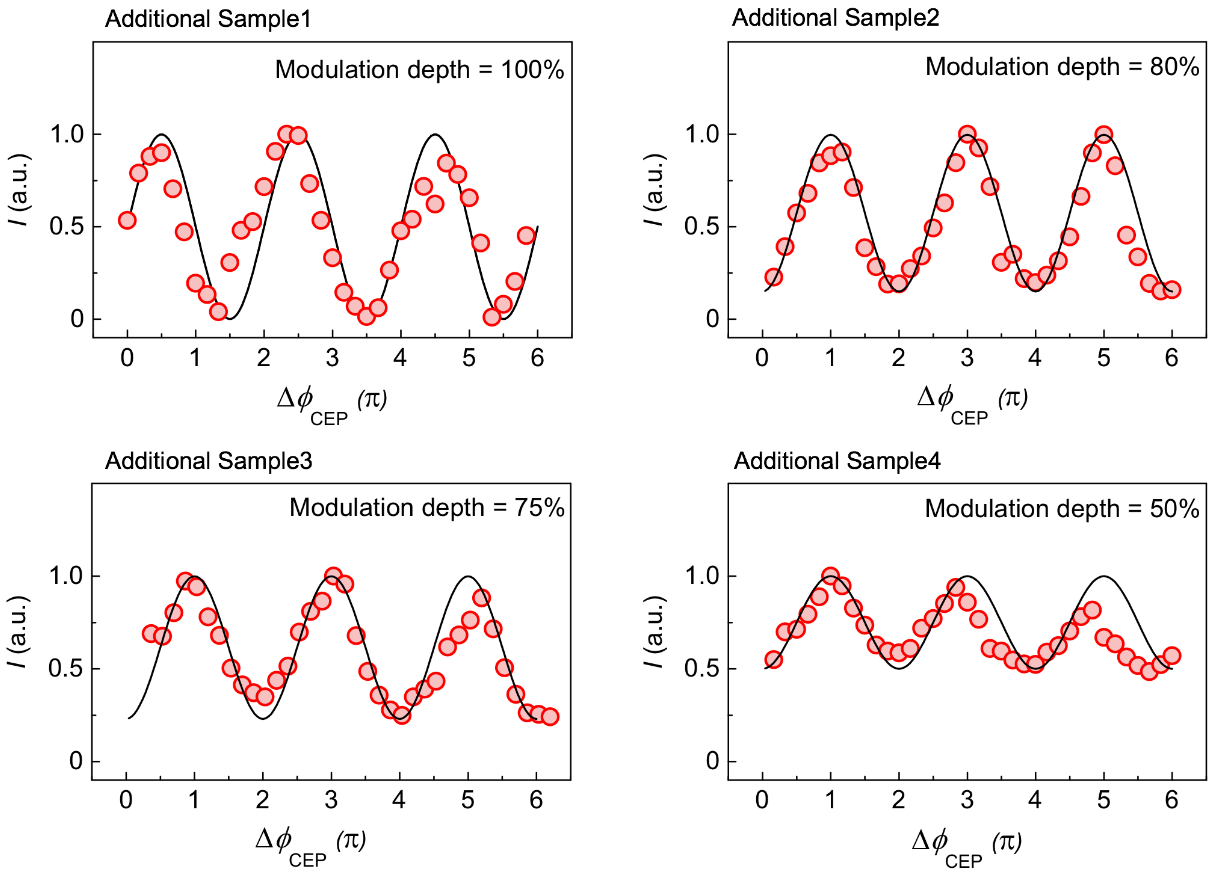


**Supplementary Figure 2.** CEP-sensitive measurement of four additional CNTs cluster emitters. All the curves show a periodical variation with CEP with high modulation depth (from 100% to 78%). Blue lines are cosine fits.

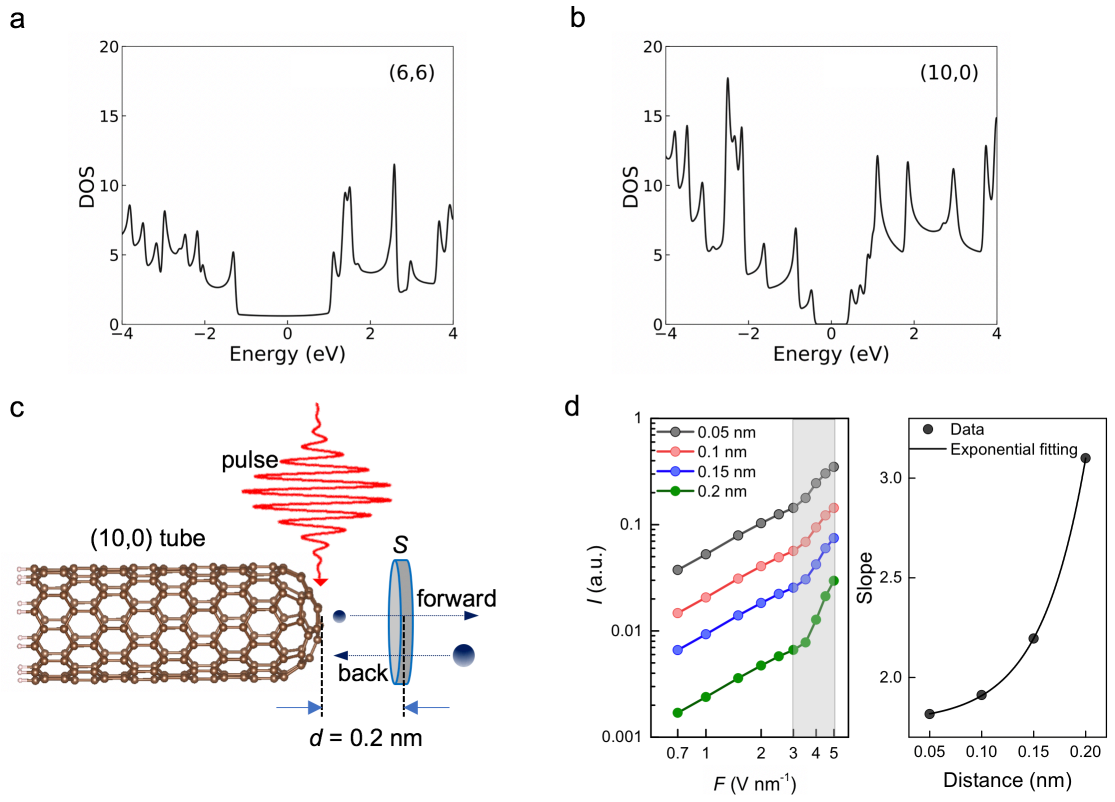


**Supplementary Figure 3. TDDFT simulation. a, b** Density of states (DOS) of the two simulated models. **c** Simulation model. Due to the absence of periodic boundary conditions in molecular calculations, it is necessary to saturate the carbon dangling bonds with hydrogen atoms, yielding a $C_{200}H_{10}$ tube. The polarization of incident light field is consistent with the tube axial. An electron detection plane is set in front of the capped end with a distance $d = 0.2$ nm, which can count the electron number crossed the plane in both vertical directions, and detect their kinetic energy. **d** Left panel, calculated *I-F* curves at different plane (S) distance (d). The slope of the curves at high field region (grey region) display a exponentially increasing with d, as shown in the right panel.

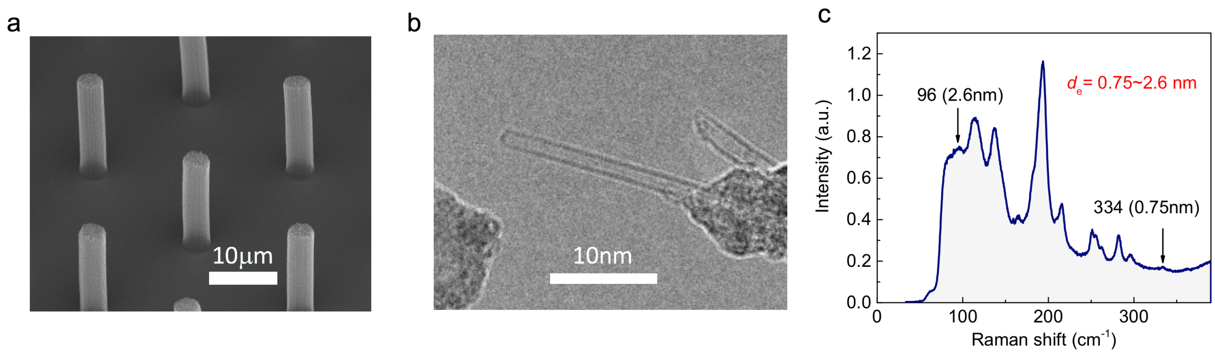


**Supplementary Figure 4. Characterization of CNTs. a** Scanning electron microscopy image of the as-grown CNT cluster array. **b** High resolution transmission electron microscopy image of an individual single-walled carbon nanotube. **c** Raman spectrum of the CNT cluster, indicating diameters ranging from 0.75 to 2.6 nm.

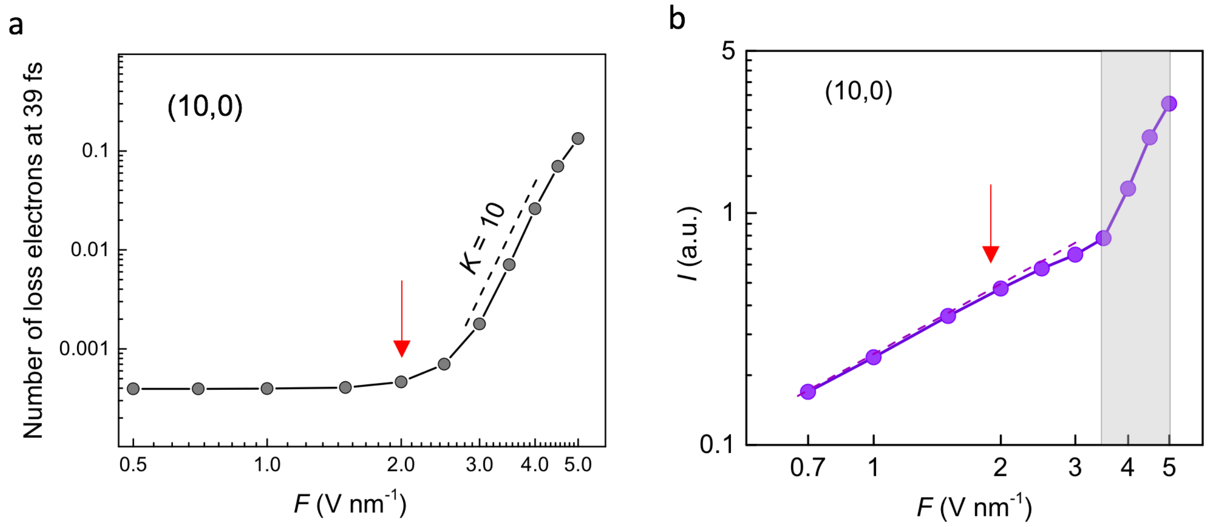


**Supplementary Figure 5. a** the number of lost electrons at 39 fs. **b** simulated *I*-*F* curve (figure 2b of the manuscript).

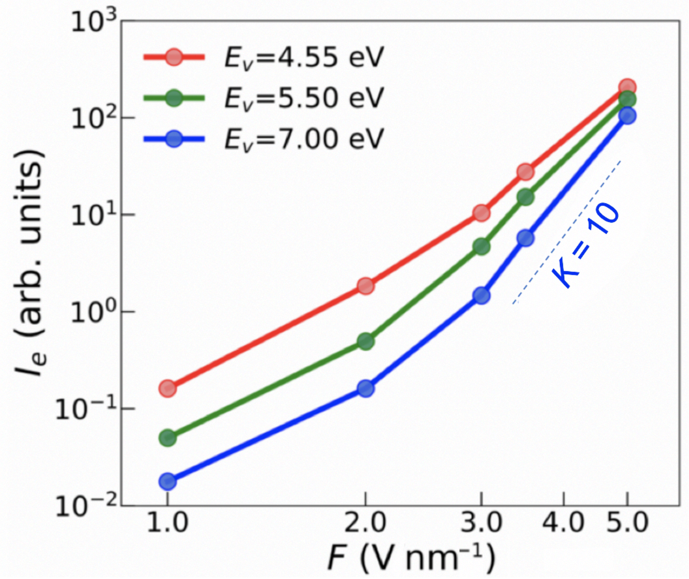


**Supplementary Figure 6**. The number of excited electron ($I_e$) above three different high energy levels ($E_V$ = 4.55 eV, 5.5 eV, 7.0 eV). A slope $K$ = 10 is obtained at $E_V$ = 7.0 eV.

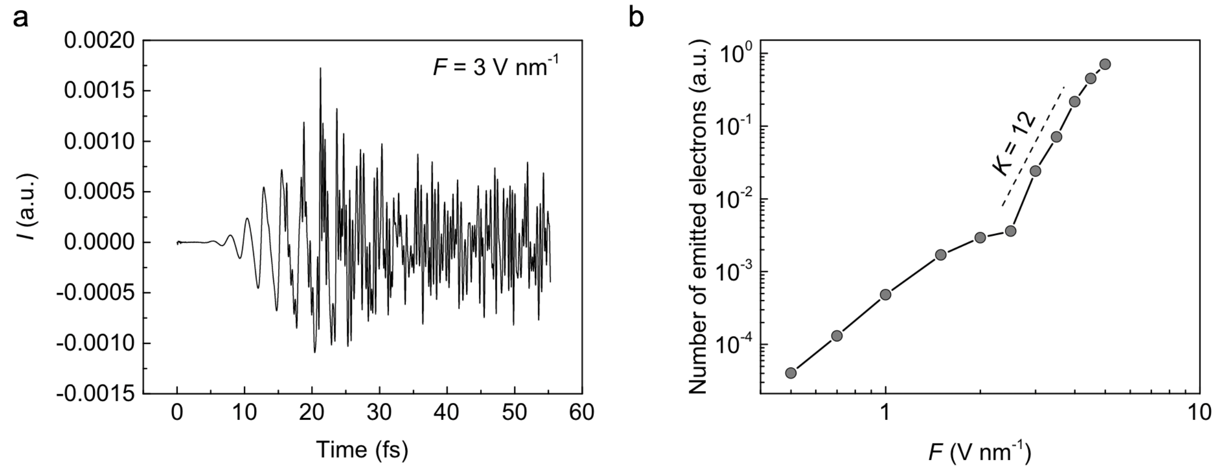


**Supplementary Figure 7. a** TDDFT calculated instantaneous current at an optical-field $F$ = 3 V nm$^{-1}$. **b** number of emitted electrons obtained by integrating the instantaneous current in **a**. A slope $K$ = 12 is obtained.

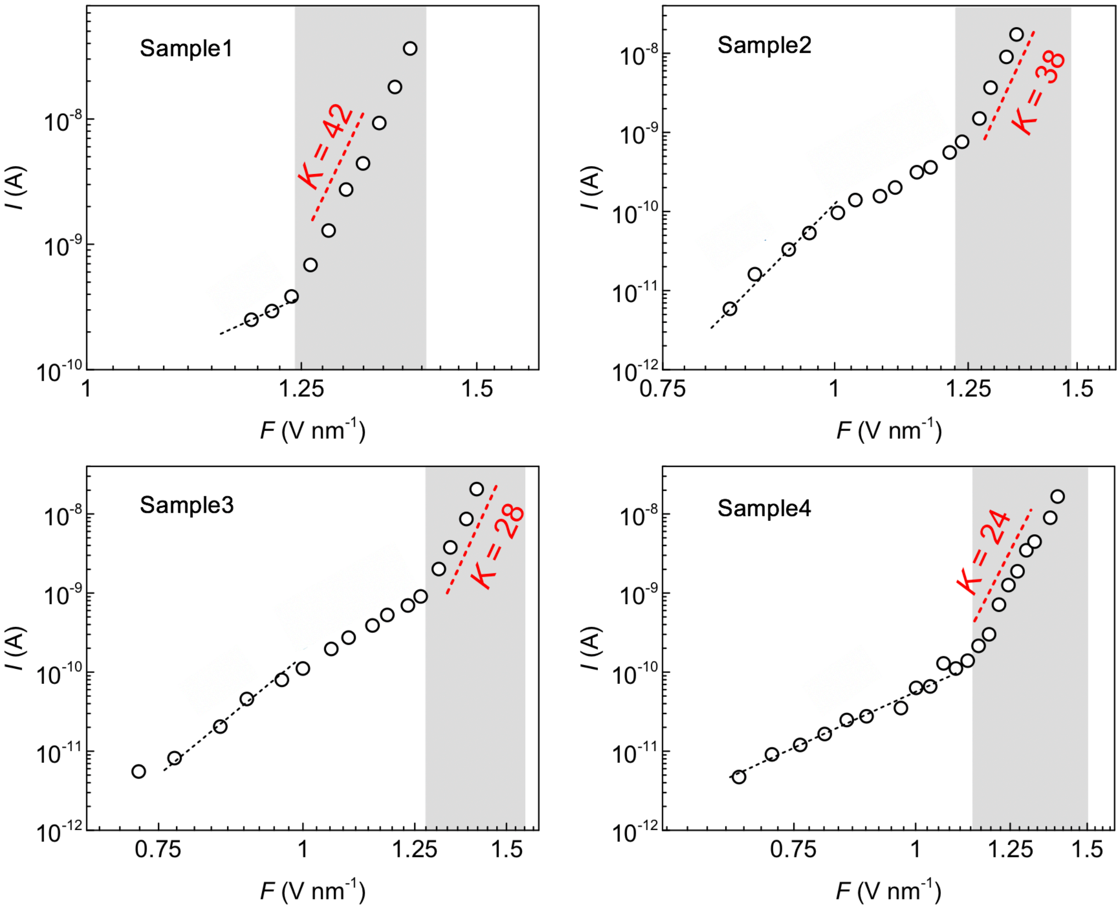


**Supplementary Figure 8.** *I-F* curves of other four different CNTs cluster emitters. All of four curves show bending up at high field region. The slope (*K*) of the curves (grey region) varies from 24 to 42.

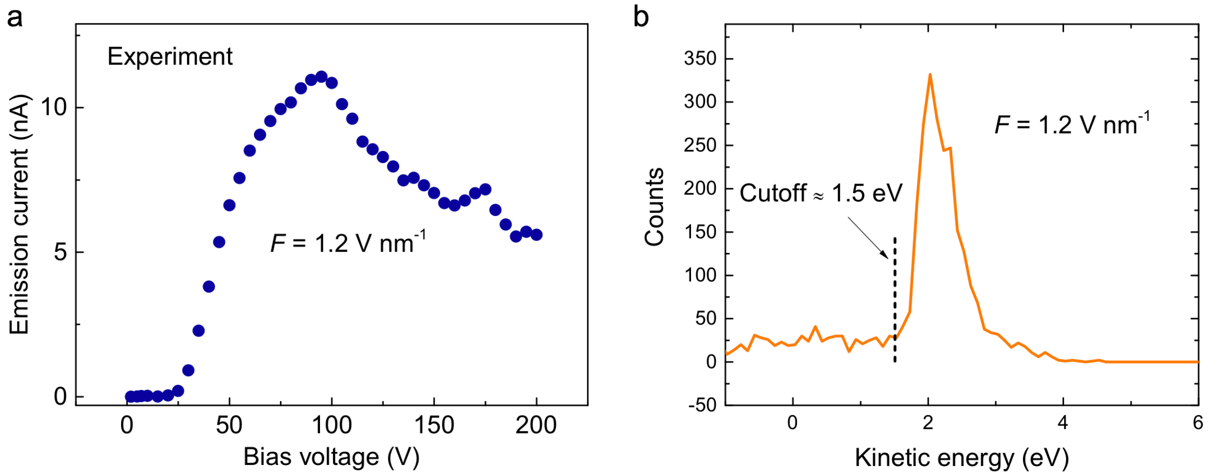


**Supplementary Figure 9. a**, dependency of photoemission current on bias voltage at fixed laser field. **b**, experimentally measured electron energy spectrum at incidence fields $F = 1.2$ V nm$^{-1}$.

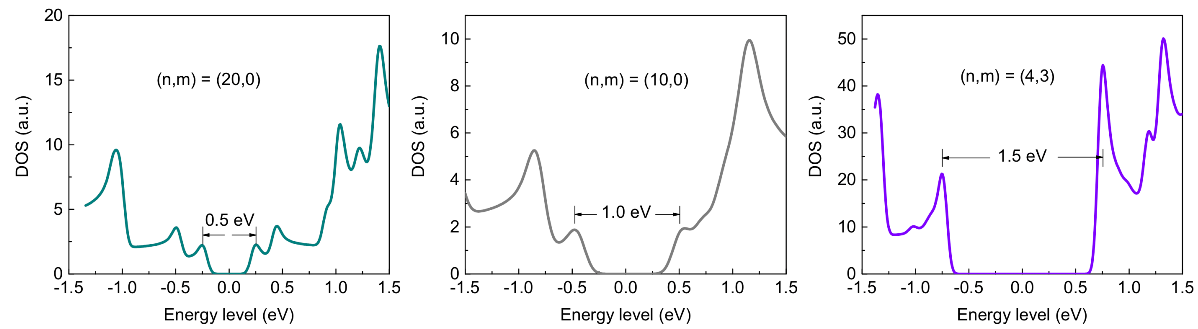


**Supplementary Figure 10.** DOS of three CNT models with different bandgaps (0.5 eV, 1.0 eV, 1.5 eV).

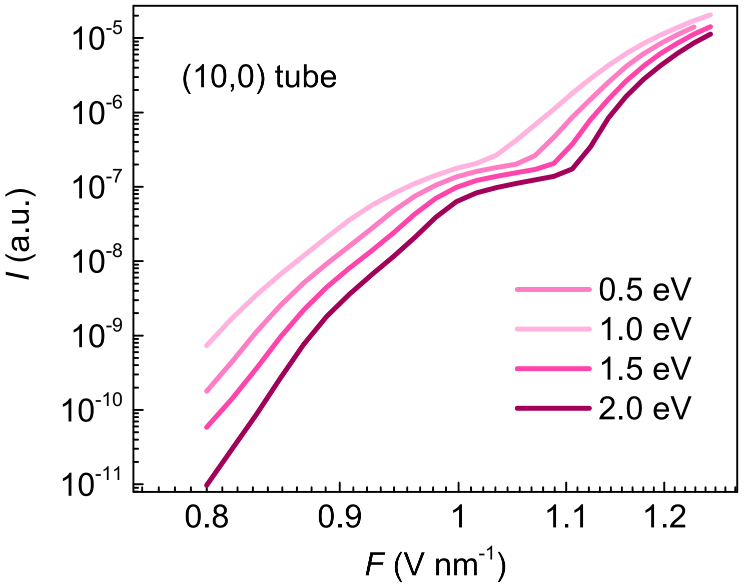


**Supplementary Figure 11**. Simulated *I-F* curves at different threshold energies ($E_{th}$ = 0.5 eV, 1.0 eV, 1.5 eV, 2.0 eV). It is noted that the nonlinearity changes with the threshold energies, while the overall trend is still maintained.

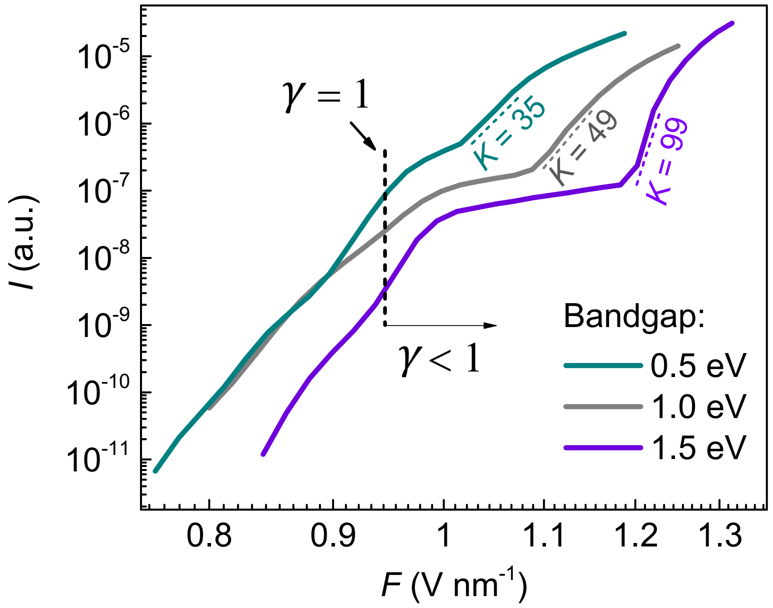


**Supplementary Figure 12**. Calculated *I-F* curves of three CNT models with different bandgaps (left: 0.5 eV, middle: 1.0 eV, right: 1.5 eV). Note that the Fowler-Nordheim model based simulation is only valid when $\gamma < 1$, which is in optical-field emission regime.

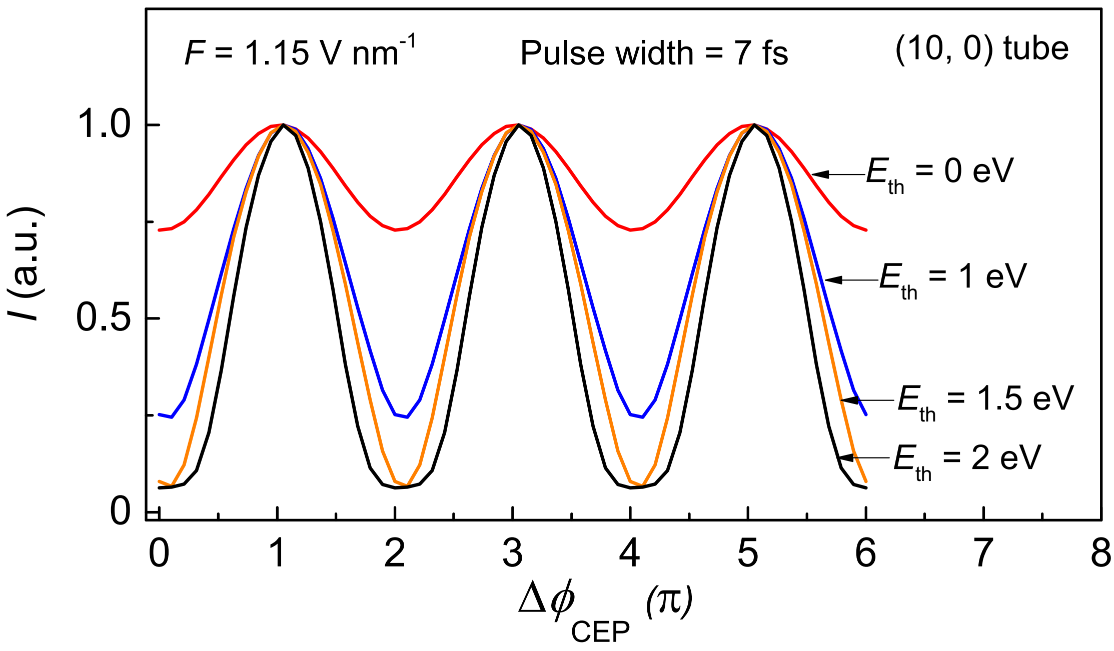


**Supplementary Figure 13**. Calculated CEP dependent emission current with different threshold energy (Eth = 0 eV, 1 eV, 1.5 eV, 2 eV). The calculation parameter: optical field F = 1.15 V nm$^{-1}$, pulse width = 7 fs. The CNT model of (10,0) tube is used.

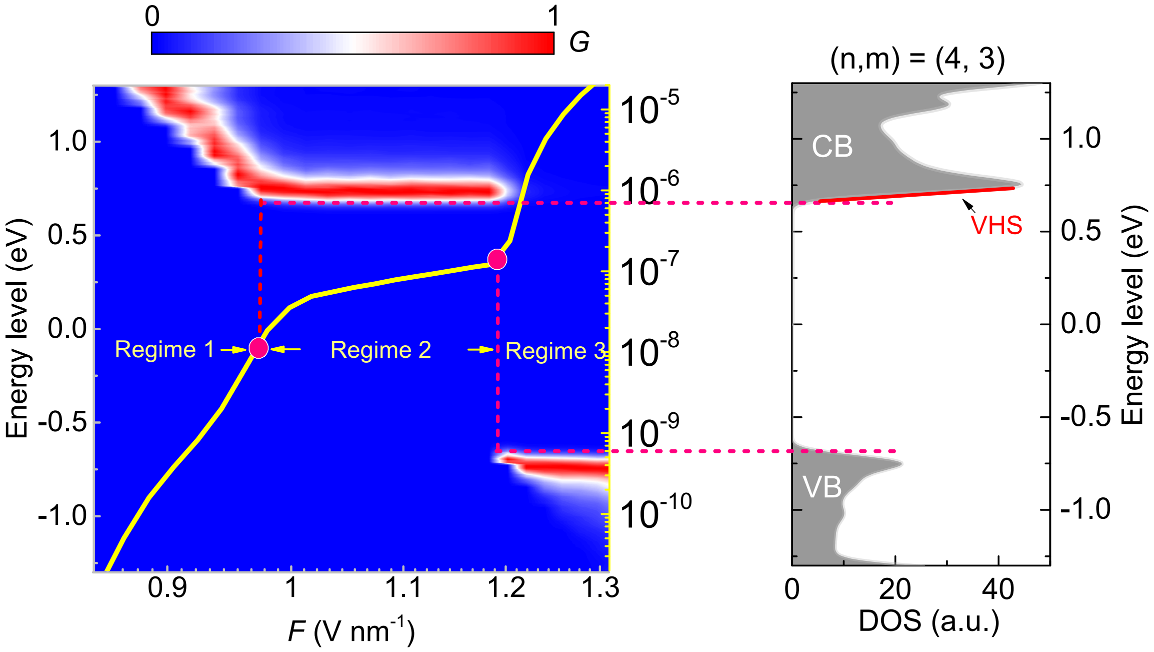


**Supplementary Figure 14**. Left panel, contour plot of the *F* dependent number (*G,* normalized at each *F* point) of emitted electrons from different energy levels, for a CNT model with a bandgap of ~1.5 eV. *I-F* curve (solid yellow line) is plotted. Right panel, DOS of the CNT model for alignment transition points. Transition points between different regimes are marked by red points, which are aligned by dashed lines to the DOS. It is obvious that the abrupt slope decreasing is due to the van Hove singularities (VHS) at the conduction band minimum (marked by red line).

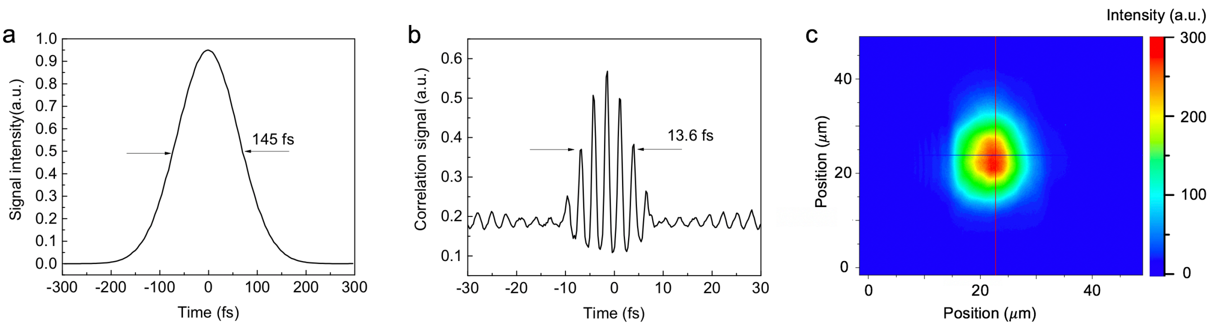


**Supplementary Figure 15. a** autocorrelation measurement of the pulse width for 100 fs laser pulse. **b** autocorrelation measurement of the pulse width for 7 fs laser pulse. **c** laser spot measurement of 7 fs laser: vertical full width half maximum is around 13.3 μm, horizontal full width half maximum is around 9.8 μm. See Supplementary Notes for details.

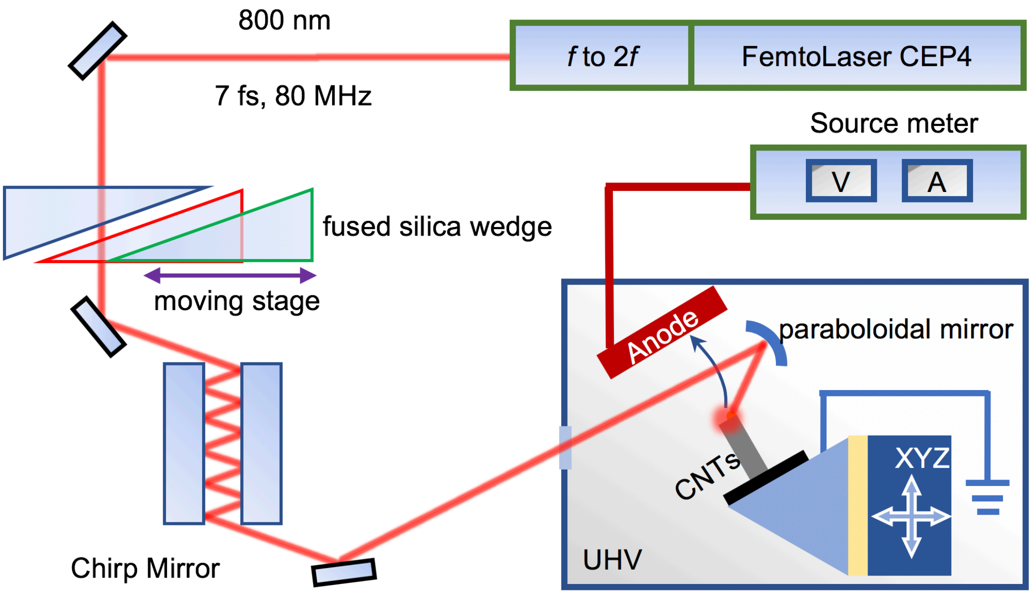


**Supplementary Figure 16. Experimental setup.** 7 fs few-cycle laser system and measurement of CEP-dependent current.

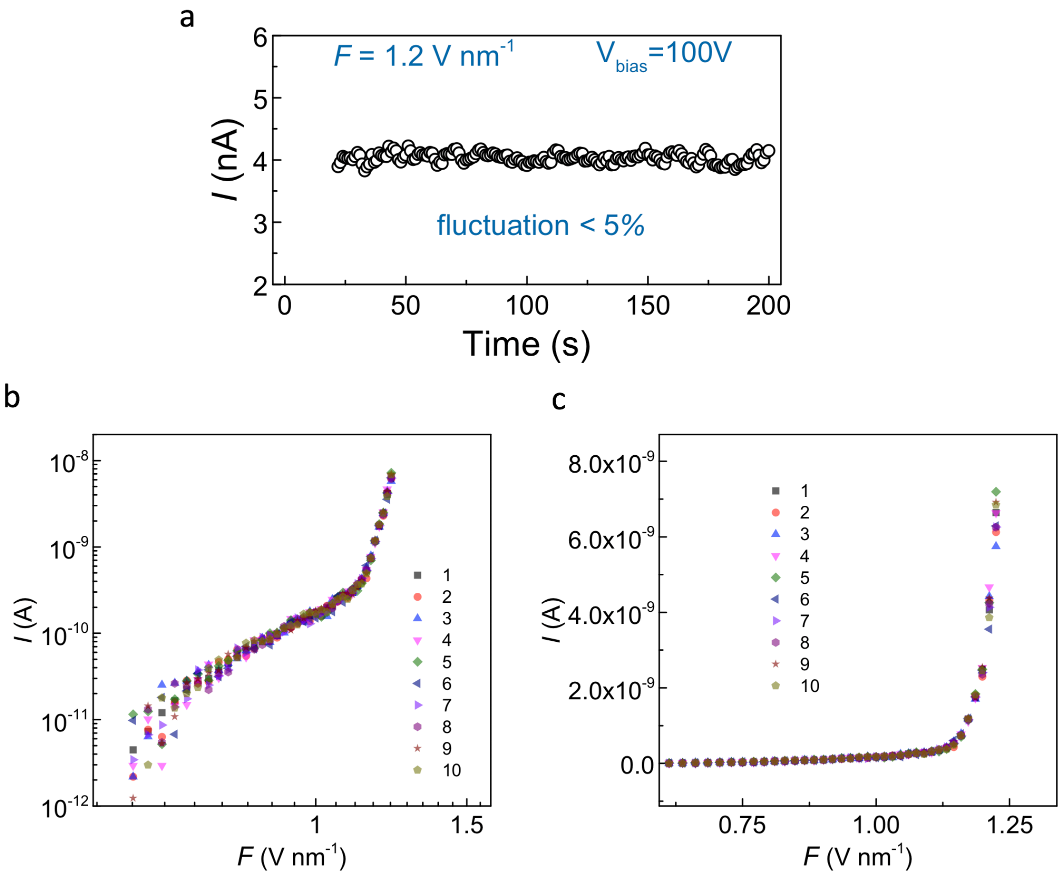


**Supplementary Figure 17. a** Stability test shows a small fluctuation < 5%. b, log-log plot of ten time cycling of *I-F* curve in Fig. 2B. **c** linear plot of ten time cycling of *I-F* curve.

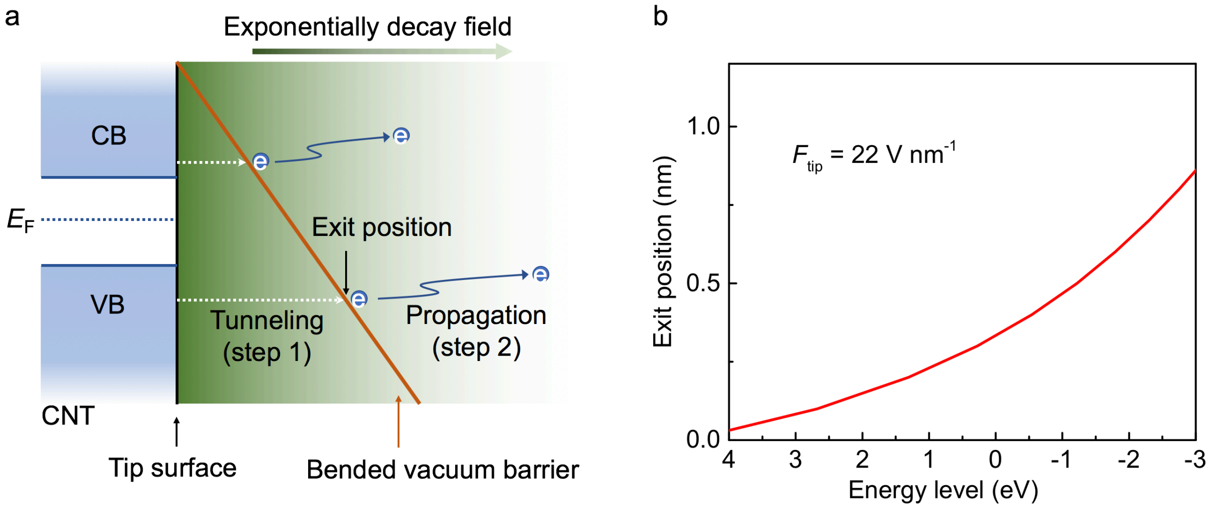


**Supplementary Figure 18. a** Diagram of the employed two-step Simpleman model. Blue shading indicates field decay. **b** Calculated dependency of tunnel exit position (distance to the tip surface) on energy level, at an enhanced optical-field of 22 V nm$^{-1}$ at the tip. It is noticed that the typical values of tunnel exit for energy level higher than -3 eV are smaller than 1nm, which is comparable to the electrons' wavelength (≈1.2 nm at 1 eV).

## 2. Supplementary Methods

**Measurement of kinetic energy spectra.** The kinetic energy spectra is measured by a time-of-flight (TOF) spectrometer with a flight tube of 45 cm length. After calibration, electrons having kinetic energies in the 3 to 50 eV range could be measured with this setup. The sample is biased at 40 V. The data recored in Supplementary Figure 9b is original data subtracted by bias voltage. For each spectrum electrons were accumulated within a measurement time of 10 seconds.

## 3. Supplementary Notes

**Supplementary Note 1: Intensity uncertainty of 100 fs laser.** The laser peak intensity (*Y*) is estimated with the measured laser parameters, i.e. the laser power (*P*), the spot size area (*A*) and the laser pulse width ($\tau$). According to the propagation of error, the overall uncertainty $\frac{U_I}{Y}$ of *Y* is calculated by the formula: $(\frac{U_I}{Y})^2 = (\frac{U_P}{P})^2 + (\frac{U_A}{A})^2 + (\frac{U_\tau}{\tau})^2$, where $U_I$, $U_P$, $U_A$, and $U_\tau$ are the standard deviations of $Y$, $P$, $A$ and $\tau$, respectively. The uncertainty of optical-field strengths (*F*) is half of $\frac{U_I}{Y}$, as $F \propto Y$.

The pulse duration is measured by a commercial autocorrelator (APE, PulseCheck 15). For example, as shown in Supplementary Figure 15a, the autocorrelation function curve has a full-width-half-maximum (FWHM) of ~145 fs, which gives that the pulse width is $\frac{145\ \text{fs}}{\sqrt{2}} \approx 103$ fs (Gaussian function fitting). According to the statistical results of the many times of measured data, the pulse width is 103 ± 5 fs.

The efficient spot size can be estimated as 2.50 μm ~ 2.85 μm in diameter by the Rayleigh criterion $\varphi = \frac{1.22\times\lambda}{N.A.}$ ($\lambda$ is the laser wavelength, *N.A.* is Numerical Aperture of objective) where laser beam is about 3 mm ~ 3.5 mm in diameter. Hence, the spot area is about 5.5 ± 0.5 $\mu m^2$.

The laser power is measured by a calibrated power meter (Thorlabs S130C) with a measurement uncertainty of 3%.

Therefore, in the present 100 fs case, the overall uncertainty of laser peak intensity is about 10.7% and the uncertainty of field strength uncertainty is about 5.4%.

**Supplementary Note 2: Intensity uncertainty of 7 fs laser.** The pulse duration is measured with a frequency resolved autocorrelator (Femtometer, Femto Laser). For example, as shown in Supplementary Figure 15b, the autocorrelation function curve has a full-width-half-

maximum (FWHM) of ~13.6 fs, which gives that the pulse width is $\frac{13.6}{2}$ fs = 6.8 fs (Lorentzian function fitting). According to the statistical results of many times of measured data, the pulse width is 6.8 ± 1 fs.

The laser spot is measured by a CCD, as shown in Supplementary Figure 15c. Fitting the data with a Gaussian-shaped intensity profile at the focus, we extract the vertical full width half maximum of the Gaussian waists = 9.8 μm, and horizontal full width half maximum = 13.3 μm. Thus, the effective spot area is about 102 $\mu m^2$. According to the statistical results of many times of measured data, the spot area is 102 ± 11 $\mu m^2$.

The laser power is measured by a power meter (Thorlabs S130C), which gives an uncertainty of 3%.

Therefore, in the present 7 fs case, the overall uncertainty of laser peak intensity is about 15.0 % and the uncertainty of optical-field strength is about 7.5 %.

## 4. Supplementary Discussion.

**Supplementary discussion of the TDDFT simulation results.** Beside the standard method described in the main text, we have tried three other counting methods to counting the photoemission current.

First, the total emitted electron may be estimated by counting the lost electron of the whole simulation system ($C_{200}H_{10}$) after the duration of the laser field. In optical-field-driven regime, the electrons may be accelerated by the optical-field to leave the emission site. The lost electron means the electron has been driven to reach the boundary and been absorbed by the boundary. If the electron still in the system after the duration of the laser field, that means it cannot leave the system. This counting method is only eligible in optical-field-driven regime, as in photon-driven regime the electrons are not driven by optical-field and hard to reach the boundary. As shown in Supplementary Figure 5a, the number of lost electrons at 39 fs (the duration of the laser field has already finished), are counted. At high field region ($F$ > 2 V $nm^{-1}$), the number of lost electrons increases greatly, which suggests the photoemission accesses optical-field-driven regime. This is consistent with the indication of optical-field-driven regime, that the simulated $I$-$F$ curves bending down at $F$ > 2 V $nm^{-1}$, as shown in Supplementary Figure 5b. The disadvantage of this method is that emission from other site of the system may be also counted, such as tail of the CNT.

Second, the number of emitted electrons may also be estimated by the counting the number of excited electrons at high energy level above vacuum level (work function) after the duration of laser field. As shown in Supplementary Figure 6, we have try the situation of three different

energy level ($E_V$ = 4.55 eV, 5.5 eV, 7.0 eV). The slope increases to 10, when we choose a high energy level of 7.0 eV. The disadvantage of this method is that high energy electrons at other site of the system may be also counted, including the tube body.

Third, the number of emitted electrons can be estimated by subtracting the electrons went back through the detection plane from the electrons went forward through the detection plane, as shown in Supplementary Figure 3c. This is achieve by integrate the instantaneous current as shown Supplementary Figure 7a. The obtained number of emitted electron is shown in Supplementary Figure 7b. A slope of 12 is obtained. The same as first method, this method is more eligible in optical-field-driven regime.

**Supplementary discussion of Simpleman model calculation.** The simulation *I-F* curves with four different threshold energies ($E_{th}$ = 0 eV, 1 eV, 1.5 eV, 2 eV ) are also shown in Supplementary Figure 11. It is noted that the nonlinearity changes with the threshold energies, while the overall trendency is still maintained. The simulation CEP-sensitive emission current with four different threshold energies ($E_{th}$ = 0 eV, 1 eV, 1.5 eV, 2 eV ) are also shown in Supplementary Figure 13. The CEP sensitivity increases with the increasing of threshold energy. The modulation depth changes from 55% to 100%.

**Supplementary Discussion of charge interaction.** The charge interaction may strongly affect the electron dynamic, such as cut-off of low kinetic energy electrons and spectra broadening. The cut-off of low kinetic energy electrons is confirmed by Supplementary Figure 9b. However, the spectra broadening is not prominent. Although we admit this is an open question that requires further study, the we try to speculate the reason as follows. The cut-off of kinetic energy electrons mainly occurs in the very local area near the tip surface (a few nm) and in an ultra-short time (a few fs). In this temporal-spatial limit, the broadening of kinetic energy is considerably small due the ultra-short time interaction. The spectra broadening mainly occurs in far field, and is a relatively long-time interaction. However, this can largely be overcome using a ultra-sharp nanotip with radially diverging particle trajectories and a large static (lightning rod) field enhancement for rapid charge escape, which is right the case of the CNT emitter used in the present work.
